# Supplementary material for: Feasibility and acceptability of a pilot, peer-led HIV self-testing intervention in a hyperendemic fishing community in rural Uganda
Source: PLoS One. 2020 Aug 7;15(8):e0236141. doi: 10.1371/journal.pone.0236141 (PMC7413506; doi:10.1371/journal.pone.0236141)
Supplement: S1 Study tool — (DOC) [file pone.0236141.s002.doc]

IMPLEMENTING A NETWORK-BASED, PEER-LED HIV SELF-TESTING INTERVENTION AMONG YOUNG PEOPLE AND ADULT MEN IN KASENSERO FISHING COMMUNITY, RAKAI DISTRICT

**BASELINE QUESTIONNAIRE – ENGLISH VERSION**

| **IDENTIFICATION OF THE PARTICIPANT** | |
| --- | --- |
| **INTERVIEWER CODE** | |  |  |  | | --- | --- | --- | |
| **INTERVIEWER INITIALS (USE SURNAME FIRST – E.G. MJ FOR MUKASA JOHN)** | |  |  | | --- | --- | |
| **INTERVIEW VENUE: __________________________________________________________**  **(One of two participating health facilities)** | |
| **STUDY COMMUNITY: __________________________________________________________** | |
| **PARTICIPANT NUMBER e.g. B01/001/YM/NS (01=community code, 001=participant’s number; YM=young male, YF=young female, AM=adult male) while NS represents the ‘interviewer initials’. ‘B’ stands for ‘baseline’. This will change to ‘F’ at follow-up.**  **Community codes: 01 – Kasensero landing site; 02- Kyebe; 03 – Gwanda**  INSERT SCREENING ID NUMBER FROM SCREENING TOOL | |  |  |  |  |  |  |  |  |  |  | | --- | --- | --- | --- | --- | --- | --- | --- | --- | --- |   **Screening ID Number**  I____I____I/I____I____I____I |
| **DATE** | |  |  |  | | --- | --- | --- |   **dd/mm/yyyy** |
| **SEX**  Male ……………………………………………………………………1  Female………………………………………………………………….2 | |  | | --- | |
| **FINAL DISPOSTION**  Interviewed ……………...........................………..……………….1  Refused………..............................................................................2  Other (specify_________________________________)…..…...3 | |  | | --- | |

**INTRODUCTION**

| **Mr/Ms _____________________ (DO NOT WRITE THE NAME, JUST MENTION)**  As mentioned in the form you have signed, I am called ________________ and am here on behalf of Makerere University School of Public Health. I am part of a team that is collecting data on HIV self-testing among young people and adult men in this district. We very much appreciate your participation in this survey. This information will help the government to plan for the scale-up of HIV self-testing interventions among people living in fishing communities. The interview will take around 1 hour. Whatever information you provide will be kept strictly confidential and will not be shown to any other unauthorized person.  May I begin the interview now?  Respondent agrees to be interviewed…….1 respondent does not agree to be interviewed…….2 |
| --- |

**INTERVIEW STARTED AT: ______AM/PM**

**100. GENERAL CHARACTERISTICS OF THE RESPONDENTS**

| **No.** | **QUESTION** | **CODING CATEGORIES** | **SKIP** |
| --- | --- | --- | --- |
|  | How old were you at your last birthday? | AGE IN COMPLETED YEARS  DON’T KNOW…………….98 |  |
| 102 | Have you ever attended school? | YES…………………………………….……….……………….…..1  NO…………………………………………..…………………..…...2 | **If 2 GOTO Q105** |
| 103 | Are you still in school? (***Being in school here refers to attending formal classes (e.g. P1 or S1 )excluding vocational studies)*** | YES……………………………………………………………….…..1  NO…………………………………………………………………….2 | **If 1, GOTO Q105** |
| 104a | What is the highest level of school you attained?  *Primary, `O` level, `A level, university or other tertiary?* | PRIMARY (P.1 –P.7).………………………………………..……..1  PRIMARY PROFESSIONAL ………….………………..………...2  O’ LEVEL (S.1 – S.4) ….……….……………………….………….3  O’ LEVEL PROFESSIONAL ….……….………………..………..4  A’ LEVEL (S.5 – S.6) ……….……..…………………..…….….…5  UNIVERSITY………….……………………………….………..…..6  OTHER TERTIARY (AFTER S.6) ……………….…………….…7  OTHER (Specify) ____________________________________8 |  |
| 104b | How long has it been since you left school? | LESS THAN A YEAR ………………………………………………1  1 YEAR AGO……………………………………..………………….2  2 YEARS AGO……………………………………..………………..3  3 YEARS AGO……………………………….…..………………….4  4+ MORE YEARS AGO…………………………………………….5  DON’T KNOW………………………………………………………..98 |  |
| 104c | What are some of the main reasons you left school? (**Select all that apply**) | Yes No  LACK OF SCHOOL FEES, UNIFORM OR MATERIALS ……..1 2  GOT MARRIED………………………………………………….....1 2  GOT SICK…………………………………………………………..1 2  NEEDED OR WANTED TO EARN MONEY…………………….1 2  NOT A GOOD STUDENT/FAILED IN SCHOOL……………......1 2  NOT INTERESTED IN SCHOOL…………………………………1 2  OTHER REASON ………………………………………………….1 2  (Specify ________________________________________________) | **Skip to Q105a** |
| 105 | If in school, in which class are you? (***Indicate actual class if below S6; otherwise, code 88 for University or 89 for other tertiary level***) | CLASS  UNIVERSITY …………………………………………….……………. 88  OTHER TERTIARY ……………………………………………………89 |  |
| 105a | Are you able to read and write in your local language? | YES ……………………………………………………………………...1  NO …………………………………………………………………….….2 | **If 2, GOTO Q106** |
| 105b | If yes, please read the following text: “A*bantu abamu balina emputtu. Bw’omugamba okukola ekintu, akuddamu akudaala. Awo naawe n’osalawo okumuleka*”  **Interviewer**: Has the participant been able to read the text? | YES, BUT WITH DIFFICULTY………………………………….….…...1  YES, WITH EASE (PROPER PRONOUNCIATION) …………………2  NO, HE/SHE WAS NOT ABLE TO READ THE TEXT……..…………3 | **IF 3, ask Q105a again and code accordingly** |
| 106 | Who do you live with? | ALONE …………………………………………………………….1  FRIENDS…………………………………………………………..2  MOTHER ALONE…………………………………………………3  FATHER ALONE……………………………………………….....4  BOTH PARENTS……………………………………………….....5  WITH OTHER RELATIVES……………………………………. .6  SPOUSE……………... …………………………………………. 7 |  |
| 107a | Are you currently married? *(by marriage I mean religious, traditional, civil or consensual union)* | NEVER MARRIED………….…………………...........................1  IN RELATIONSHIP BUT NOT MARRIED….............................2  MARRIED/UNION…………………………………………..……..3  DIVORCED/SEPARATED…………….……..............................4  WIDOWED…………………..……………..….............................5 | **IF 1,4,5 GOTO Q108**  **IF 2, skip to Q107c** |
| 107b | In which type of marriage are you currently in? | MONOGAMOUS……………………………….……………...…1  POLYGAMOUS……….………………………...………………..2 |  |
| 107c | How old is your partner? **INTERVIEWER NOTE:** ***If not married but in a relationship, still ask about the most recent sexual partner’s age*** | AGE IN YEARS  DON’T KNOW 98  Not Applicable 97 |  |
| 108 | What is your religion? | CATHOLIC…………………………………………....….........………...1  ANGLICAN/PROTESTANT……………….……...............……………2  MOSLEM………………………………………………...............………3  PENTECOSTAL /BORN AGAIN / EVANGELICAL…........................4  SEVENTH DAY ADVENTIST……….………………………................5  ORTHODOX…………………………………………………………..….6  OTHERS(specify)………………………………………......……...........7 ________________________________________ |  |
| 109 | What is your main occupation, where you spend most of your time? | FISHING……………………………………………………………….…1  FISHING-RELATED ACTIVITY (E.G. NET REPAIRING)…………..2  SEX WORKER…………………………………………………………..3  PEASANT FARMER…………………..………..……....……………...4  SALARIED…………………………………………..…….……………..5  BUSINESS/COMMERCIAL……………………..………………….….6  CASUAL WORKER………………………..…………………………...7  HOUSE WIFE…………………...........………….………………….….8  PUPIL/STUDENT/NO OCCUPATION....……………………………. 9  OTHER (SPECIFY)………………………………..…………..….……10 ______________________________________________ | **IF8,9 GOTO Q111** |
| 110 | What is your estimated average monthly expenditure? | LESS THAN 10,000/=………………..…….………………………..…1  10,000-30,000/=………………………………………………………...2  31,000-50,000/=………………..………………………..…….......…...3  51,000-100,000………………………….……..….………………...….4  101,000-500,000……………………………...……………….…..…...5  >500,000……………………………………………………………...…6 |  |
| 111 | Have you ever had a sexually transmitted disease? | YES……………………………………………………………………...1  NO…………………………………………………………………....….2 | **IF 2, GOTO**  **Q115** |
| 112 | Did you seek treatment for the sexually transmitted infection (STI)? | YES…………………………………………………………….………..1  NO……………………………………………………………………….2 |  |
| 113 | How soon did you seek treatment after realizing that you had any STI? | SAME DAY ……………………………………………………………..1  1-2 DAYS ……………………………………………..………………..2  3-5 DAYS……………………………………………….………………3  1 WEEK………………………………………………….……………..4  MORE THAN 1 WEEK…………………………………….………….5 |  |
| 114 | From where did you seek treatment? | SHOP………………………………………………….……….……….1  PHARMACY………………………………………………….….....…..2  GOVT. HOSPITAL/HEALTH CENTRE/CLINIC……………..……..3  PRIVATE DOCTOR/NURSE/CLINIC……….………...…………….4  HERBAL/ TRADITIONAL PROVIDER …………………………… ..5  OTHER, **SPECIFY**: ____________________________________6 |  |
| 115 | Do you own a mobile phone? | YES……………………………………………………………………...1  NO……………………………………………………………………….2 | **IF 2, GOTO Q201** |
| 116 | If yes, what type of phone do you have? | ORDINARY PHONE……………………………………………………………1  SMART PHONE WITH TOUCH SCREEN………………………………….. 2  OTHER TYPE (SPECIFY) ____________________________________ 3 |  |

**200. SEXUAL BEHAVIOUR**

Now I am going to ask you questions about sexuality and some questions will be about your own private life. Some of these questions need to be rather detailed and personal. Since this survey is confidential and your name is not included on this paper, no one else will know or connect your answers with you. We would appreciate your participation in answering these questions as openly as possible.

| No. | QUESTION | CODING CATEGORIES | SKIP |
| --- | --- | --- | --- |
| 201 | Have you ever had any sexual intercourse in your life? | YES ……………………………………....1  NO ………………………………….….....2 | **IF 2,**  **GOTO Q301** |
| 202 | If yes, how old were you when you had sexual intercourse for the very first time? | AGE IN YEARS ……………….  DON’T KNOW…..………….. 98 |  |
| 203 | Which person did you have sex with for the first time? | Boyfriend/girlfriend….………………………….1  Husband/wife….………………………………...2  Stranger………………………………………….3  Brother/sister……………………………………4  Teacher…………………………………….….....5  Uncle/Auntie……………………………………..6  Father.……………………………………………7  Sex worker…………………………….…………8  Fish monger/fish trader ………………………..9  Other relative (specify)_________________..10  Other (Specify) _____________________.....11 |  |
| 204 | How old was the partner that you had sex with for the first time? | Same age as me ……………………………….1  Younger than me ……………………………….2  1-2 years older than me………………………..3  3-4 years older than me………………………..4  5 or more years older than me………………..5  Don’t know………………………………………98 |  |
| 205 | The first time you had sexual intercourse, were you under the influence of alcohol or drugs? | YES …………………………………………… 1  NO……………………………………………….2  Don’t know/Don’t Remember…………………98 |  |
| 206 | When was the last time you had sexual intercourse? | Within 1 week………………………..……..…….1  Within 1 month………………………….……..….2  >1 but <3 months …………………….……..…...3  >3 months ago………..……..……………….…...4 | **IF 4**  **GOTO Q213** |
| 207 | The number of sexual partners people have may differ from person to person. Some people report having had one sex partner, some 2 or more partners. In the **PAST THREE MONTHS**, how many different partners have you had sex with? | _____________Partners |____|____|  DON’T KNOW 98 |  |
| 208 | In the past three months, how often did you use condoms with all these partners? | Always…………………………………………...1  Sometimes ……………………………………...2  Rarely…………………………………………….3  Never …………………………………………….4 |  |
| 209 | What was your relationship with the person you most recently had sex with? | Boyfriend/girlfriend….………………………….1  Husband/wife….………………………………...2  Stranger………………………………………….3  Brother/sister……………………………………4  Teacher…………………………………….….....5  Uncle/Auntie……………………………………..6  Father..……………………………………………7  Sex worker…………………………….…………8  Fish monger/fish trader ………………………..9  Other relative (specify)_________________..10  Other (Specify) _____________________.....11 |  |
| 210 | How old was the partner that you had sex with for the last time? | Same age as me ………………………………. 1  Younger than me ………………………………. 2  1-2 years older than me ………………………..3  3-4 years older than me ………………………..4  5 or more years older than me ………………..5  Don’t know ……………………………………. 98 |  |
| 211 | Thinking of **THE LAST TIME** you had intercourse with this partner, did you (or your partner) use a condom? | YES ……………………….…………………….1  NO …………………………….………………...2 | **IF 2 GOTO Q213** |
| 212 | Thinking of all the times you had intercourse with this partner **IN THE LAST 3 MONTHS**, would you say you used a condom all the time, sometimes, or never? | Never……………………………..……………...1  Sometimes ………………….……..….....….…..2  Always …………………..……………………....3  NA (Had no sex in last 12 months)………...…4  Don’t know/don’t remember ……….………....98 |  |
| 213 | Some people are worried about sexually transmitted infections, including HIV. How concerned are you or were you about getting an STI/HIV from your partner when you had sex with them? | Very concerned …………………………………1  Somewhat concerned ………………………….2  Not really concerned …………………………...3  Not at all concerned ……………………………4  Don’t know ……………………………………..98 |  |
| 214 | Are you currently using a condom? | YES ………………………………………….….1  NO ………………………………………….…...2 | **IF 2 GOTO Q217** |
| 215 | If you are currently using a condom; what is the reason for using a condom? | Family planning……………….…………….…..1  HIV/other STI prevention………………...…….2  Both ……………………….……………………..3 |  |
| 216 | Are you using a male or female condom? | Male condom……………….…………....……...1  Female condom……….…….…...……..............2  Both…………………………...……………….....3 |  |
| 217 | Do you know of a place where a person can get condoms? | YES ……………………………………………….1  NO ………………………………………………...2  DON'T KNOW .………………………………….98 |  |
| 218 | If you wanted to, could you yourself get a condom? | YES . . . . . . . . . . . . . . . . . . . . . . . . . …… . . 1  NO . . . . . . . . . . . . . . . . . . . . . . . . . …… . . 2  DON'T KNOW/UNSURE . . . . . . . . . . …... …. 98 | **If 1 – skip to Q220** |
| 219 | If you can’t get a condom or you don’t know/not sure if you can get a condom yourself, what is the main reason for this? | I fear to ask for a condom …………………….…..1  I cannot afford the cost of condoms ……….…….2  I don’t know where to find them …………..……..3  Place is very far …………………………………...4  My partner does not like condoms ………………5  My religion does not allow me to use condoms……6  Other (specify) _____________________……...7 | **If 3, please check response to Q217.** |
| 220 | In the past three months, how often did you use alcohol before you had sex with your most recent sexual partner? | Never…………………. ………………………..1  Some of the time………………………….……2  Most of the time…………………………….….3  Always……………………………………….….4  No partner ……………………………………...5 |  |
| 221 | At times or routinely, some people do have sex in exchange for gifts, money, or services.  In the last 3 months, did you have sex where you received something in exchange including gifts, money or services from any sexual partner? | YES ………………………………..………….1  NO ……………………………………..……...2  DON'T KNOW .……………………………….98 |  |
| 222 | At times or routinely, some people do have sex in exchange for gifts, money, or services.  In the last 3 months, did you have sex where you gave something in exchange including gifts, money or services to any sexual partner? | YES ……………………………………..…….1  NO ……………………………………..……...2  DON'T KNOW .……………………………….98 |  |

**300. GENERAL HIV KNOWLEDGE, ACCEPTING ATTITUDES TOWARDS PLHIV, HIV TESTING**

| **KNOWLEDGE OF HIV/AIDS** | | | |
| --- | --- | --- | --- |
| 301 | Have you ever heard of an infection called HIV, the virus that causes AIDS? | Yes……………………………….…….1  No ……………………………………..2 | **IF 2, GOTO Q331** |
| 302 | If a man/woman has HIV, does his/her partner always have HIV? | Yes…………………………………….1  No ……………………………………..2  DON’T KNOW ……………………….98 |  |
| 303 | Is it possible for a healthy-looking person to have HIV? | Yes…………………………………….1  No ……………………………………..2  DON’T KNOW ……………………….98 |  |
| 304 | If a mother is HIV-positive, can she transmit HIV to her unborn baby? (Unprompted) | Yes…………………………………….1  No ……………………………………..2  DON’T KNOW ……………………….98 |  |
| 305 | Do you know of a place(s) where people can get tested for HIV? | Yes………………………….………….1  No ……………………….……………..2 |  |
| 306 | Can people reduce their chance of getting the HIV virus by having just one uninfected sex partner who has no other sex partners? | YES . . . . . . . . . . . . . . . . . . . . . . . . .1  NO . . . . . . . . . . . . . . . . . . . . . . …. 2  DON'T KNOW . . . . . . . . . . . . . .. 98 |  |
| 307 | Can people get the AIDS virus from mosquito bites? | YES . . . . . . . . . . . . . . . . . . . . . . . . .1  NO . . . . . . . . . . . . . . . . . . . . . . …. 2  DON'T KNOW . . . . . . . . . . . . . .. 98 |  |
| 308 | Can people reduce their chance of getting the AIDS virus by using a condom every time they have sex? | YES . . . . . . . . . . . . . . . . . . . . . . . . .1  NO . . . . . . . . . . . . . . . . . . . . . . …. 2  DON'T KNOW . . . . . . . . . . . . . .. 98 |  |
| 309 | Can people get the AIDS virus by sharing food with a person who has AIDS? | YES . . . . . . . . . . . . . . . . . . . . . . . . .1  NO . . . . . . . . . . . . . . . . . . . . . . …. 2  DON'T KNOW . . . . . . . . . . . . . .. 98 |  |
| 310 | Can people get the AIDS virus because of witchcraft or other supernatural means? | YES . . . . . . . . . . . . . . . . . . . . . . . . .1  NO . . . . . . . . . . . . . . . . . . . . . . …. 2  DON'T KNOW . . . . . . . . . . . . . .. 98 |  |
| 311 | Can the virus that causes AIDS be transmitted from a mother to her baby:  During pregnancy?  During delivery?  Through breastfeeding? | YES NO DK  DURING PREG ……. … .. 1 2 98  DURING DELIVERY…. . ...1 2 98  BREASTFEEDING … . ,,,1 2 98 |  |
| 312 | Can someone prevent her/himself from acquiring HIV/AIDS through abstinence? | YES . . . . . . . . . . . . . . . . . . . . . . . . .1  NO . . . . . . . . . . . . . . . . . . . . . . …. 2  DON'T KNOW . . . . . . . . . . . . . .. 98 |  |
| 313 | Can male circumcision help to reduce risk of acquiring HIV/AIDS? | YES . . . . . . . . . . . . . . . . . . . . . . . . .1  NO . . . . . . . . . . . . . . . . . . . . . . …. 2  DON'T KNOW . . . . . . . . . . . . . .. 98 |  |
| 314 | Are there any special drugs that a doctor or a nurse can give to a woman infected with the AIDS virus to reduce the risk of transmission to the baby? | YES . . . . . . . . . . . . . . . . . . . . . . . . .1  NO . . . . . . . . . . . . . . . . . . . . . . …. 2  DON'T KNOW . . . . . . . . . . . . . .. 98 |  |
| **ACCEPTING ATTITUDES ABOUT PEOPLE LIVING WITH HIV** | | | |
| 315 | Would you buy fresh vegetables from a shopkeeper or vendor if you knew that this person had the AIDS virus? | YES . . . . . . . . . . . . . . . . . . . . . . . . .1  NO . . . . . . . . . . . . . . . . . . . . . . …. 2  DON'T KNOW . . . . . . . . . . . . . .. 98 |  |
| 316 | If a member of your family got infected with the AIDS virus, would you want it to remain a secret or not? | YES, REMAIN A SECRET………… 1  NO . . . . . . . . . . . . . . . . .. . . . . . 2  DK/NOT SURE/DEPENDS ..……… 98 |  |
| 317 | If a member of your family became sick with AIDS, would you be willing to care for her or him in your own household? | YES . . . . . . . . . . . . . . . . . . . . . . . . 1  NO . . . . . . . . . . . . . . . . . . . . . . . . . 2  DK/NOT SURE/DEPENDS ……….. 98 |  |
| 318 | In your opinion, if a female teacher has the AIDS virus but is not sick, should she be allowed to continue teaching in the school? | SHOULD BE ALLOW ……………. 1  SHOULD NOT BE ALLOWED …… 2  DK/NOT SURE/DEPENDS ……… 98 |  |
| **HIV TESTING** | | | |
| 319 | Have you ever tested for HIV? | Yes…………………….……………….1  No ………………………….…………..2 | **IF 2**  **GOTO**  **Q328** |
| 320 | How many times have you been tested for HIV? | Number ________ |  |
| 321 | When was your last HIV test? | Less than 1 month ago …………..………….1  1-3 months ago ………………………….……2  More than 3 months ago ……………….……3  Can’t remember ………………………....……4 |  |
| 322 | Are you willing to tell me the last HIV test result you received? | Yes………………………………………..….1  No ……………………………………………2 | **IF 2**  **GOTO**  **Q324** |
| 323 | If yes, what was the result of that HIV test? | Positive…………………………….………1  negative ………………………….………..2  indeterminate……………………..………3  did not receive results ……………..…4 |  |
| 324 | Have you disclosed your HIV status to your sexual partner? | Yes……………………………….…….….1  No ………………………………………....2  No partner/not applicable……..…….3 |  |
| 325 | **Interviewer: Skip to Q328 if respondent was NOT HIV-positive at last test.**  Have you registered at a clinic for HIV care since the last time you tested HIV-positive? | Yes ………………………………..……….1  No ……………………………..…………...2 | **IF 2, SKIP TO Q328** |
| 326 | Are you currently on antiretroviral therapy? | Yes………………………………………….…….1  No ………………………………………………...2 | **IF 2 GOTO**  **Q328** |
| 327 | How long ago did you start taking ARVs to manage your HIV? | ______________Months  _______________Years |  |
| 328 | Have you asked your spouse/current partner if he/she has ever tested for HIV? | Yes……………………………….……….………….1  No ……………………………………….….………..2  HAVE NO PARTNER/NOT APPLICABLE............ 3 | **IF 3, SKIP TO Q331** |
| 329 | Do you know whether your spouse/current partner has ever tested for HIV? | Yes…………………………………………….1  No ……………………………………………..2 |  |
| 330 | Have you ever tested together with your current partner as a couple? | Yes…………………………………………….1  No ……………………………………………..2 |  |
| 331 | **Now I would like to ask you some questions about your health**. In the **past three months**, have you had a disease which you got through sexual contact? | YES . . . . . . . . . . . . . . . . . . . . . . …... . .1  NO . . . . . . . . . . . . . . . . . . . . . . …. ….2  DON'T KNOW . . . . . . . . . . . . . …….98 | **Cross-check with Q111.**  **If 2,98 – Skip to Q401** |
| 332 | The last time you had (PROBLEM FROM 331), did you seek any kind of advice or treatment? | YES . . . . .. . . . . . . . . . . ………….. . . 1  NO . . . . . . . . . . . . .. . . ………….. …2 | **IF 2 GOTO**  **Q401** |
| 333 | Where did you go?  Any other place?  PROBE TO IDENTIFY EACH TYPE OF SOURCE.  IF UNABLE TO DETERMINE IF PUBLIC OR PRIVATE SECTOR, WRITE THE NAME OF THE PLACE.  (NAME OF PLACE(S)) | **PUBLIC SECTOR**  GOVERNMENT HOSPITAL . . . . . . . .1  GOVT. HEALTH CENTER . . . . . . . .2  STAND-ALONE VCT CENTER . . . .3  FAMILY PLANNING CLINIC . . . . . 4  OUT REACH . . . . . . . . . . . . . . 5  VILLAGE HEALTH TEAM ………… 6  OTHER PUBLIC____ ______7  (SPECIFY)  **PRIVATE MEDICAL SECTOR**  PRIVATE HOSPITAL/CLINIC ……… 8  STAND-ALONE VCT CENTER …… 9  PHARMACY/DRUG SHOP . … 10  PRIVATE DOCTOR/NURSE/  MIDWIFE …………………. 11  OUT REACH . . . . . . . . . . . . . . . 12  TASO . . . . . . . . . . . . . . . . . . 13  AIDS INFORMATION CENTRE .…. 14  OTHER PRIVATE/NGO/MEDICAL _____________________________ 15  (SPECIFY)  OTHER ______________________ 16  (SPECIFY) |  |

**400 KNOWLEDGE, ATTITUDES AND PERCEPTIONS TOWARDS HIV SELF-TESTING**

| 401 | Have you ever heard of a method of HIV testing called ‘oral HIV self-testing’? | Yes…………………………………………..1  No……………………………………………2  Don’t know/not sure……………………….98 | | | | **If 2,98 – skip to preamble before Q403a** |
| --- | --- | --- | --- | --- | --- | --- |
| 402a | If yes, where did you hear about oral HIV self-testing/where did you get information about oral HIV self-testing? | Radio/TV  Newspaper  Community health mobilizer  Health facility within the area  Rakai Health Sciences Program  Village meeting  Community outreach  Other source (specify)___________________________ | | Yes No  1 2  1 2  1 2  1 2  1 2  1 2  1 2  1 2 | |  |
| 402b | If yes, what have you heard about oral HIV self-testing? (***Circle all that apply***) | HIV self-testing can be done outside formal health facilities  HIV self-testing does not use blood to detect HIV  HIV self-testing uses “saliva” to detect HIV  HIV self-testing is easy to perform  HIV self-testing yields accurate results  Other (specify) _____________________________________ | | Yes No  1 2  1 2  1 2  1 2  1 2  1 2 | |  |
|  | **INTERVIEWER: PLEASE LEVEL THE PARTICIPANT’S UNDERSTANDING OF HIV SELF-TESTING**  **Oral HIV self-testing is a procedure in which an individual collects an oral swab (from the mouth) using a kit (i.e. HIV self-test kit), places the kit in a testing vial (bottle) and waits for 20 minutes to read and interpret the HIV test results. It works more or less in the same way as a pregnancy test kit. HIV-positive results are shown with two red lines on the screen of the kit while HIV-negative results are shown with one line. If no lines show up on the screen, the test is considered to be invalid.** | | | | |  |
| 403a | Would you be willing to perform HIV self-testing, if the HIV self-test kits were made freely available to you? | Yes……………………………………………….….………..1  No…………………………………………………..…………2  Don’t know/not sure…………………………….………….98 | | | |  |
| 403b | If HIV self-test kits became available, where would you like to pick them from? (***Circle all that apply***) | Yes No  Hospital/health center/clinic 1 2  Pharmacy 1 2  Drug shop 1 2  Community-based distributor’s place 1 2  Village health team member’s place 1 2  HIV counsellor/Expert client 1 2  Church/mosque 1 2  Other place (Specify)_________________________ 1 2 | | | |  |
| 403c | If HIV self-test kits became available, what kind of support would you need in order to perform the test without making errors? (***Prompted question; circle all that apply***) | Yes No  How to obtain the oral swab ……………………………1 2  How to perform the test itself……………………………1 2  How to read the results…………………………………..1 2  How to interpret the results………………………………1 2  Pre- and post-testing counselling……………………….1 2  Referral for HIV care if HIV-positive…………………….1 2  How to dispose of the kit after use……………………...1 2  Other support (Specify) ________________________1 2 | | | |  |
| 404a | If HIV self-test kits were to be freely distributed to people in this community, what kind of person would you prefer to distribute the kits? (***Circle all that apply***) | Yes No  Trained community health volunteer 1 2  Friend/relative 1 2  Sexual partner (spouse/boyfriend/girl-friend) 1 2  Local council official 1 2  Religious official 1 2  Other (specify)______________________________ 1 2 | | | |  |
| 404b | If HIV self-test kits became available, would you mind if the HIV self-test kits distributor were of the opposite sex? | Yes No  Yes, I would mind …………………………………………. 1 2  No, I would not mind ……………………………………….1 2 | | | |  |
| 405 | Please indicate if you strongly disagree, neither agree nor disagree or strongly agree with the statements shown on the right. | **1=Strongly disagree 2=Neither agree nor disagree 3=Strongly agree** | | | |  |
|  |  | If HIV self-test kits became available, I will be glad to get one to use myself  HIV self-test kits will promote promiscuity in the community  People in the fishing communities do not like to test for HIV  People in the fishing communities will not like to use HIV self-test kits even if they became available free of charge  HIV self-test kits will increase the number of people who are aware of their HIV status  If well trained, I feel that I can confidently use the HIV self-test kit to test for HIV  People will think that those who use HIV self-test kits are promiscuous  I don’t think the HIV self-test kit can yield correct HIV test results  I would rather use the existing HIV testing approaches than use the HIV self-test kit  Most people in my community will embrace the use of HIV self-test kits | | | 1 2 3  1 2 3  1 2 3  1 2 3  1 2 3  1 2 3  1 2 3  1 2 3  1 2 3  1 2 3 |  |
|  | **INTERVIEWER: PLEASE INTRODUCE THE PROPOSED HIV SELF-TESTING STUDY HERE.**  **We plan to conduct a study in which we intend to distribute HIV self-test kits to selected members in this community. The distribution will be done by local people in your community who will be selected and trained for this purpose. The local distributors will teach potential users how to use the kits and how to interpret the results.** | | | | |  |
| 406a | Would you be willing to receive kits from a trained local person in your community? | Yes……………………………………………………….1  No………………………………………………………...2  Don’t know/not sure…………………………………….98 | | | | **IF 2,3 – SKIP TO Q406e** |
| 406b | If yes, where would you like the HIV self-test kits distribution event to take place? (***Circle all that apply***) | Yes No  Own home 1 2  Own work-place 1 2  Local distributor’s work place 1 2  Anywhere within the community but not at home 1 2  Home of local HIV self-test kit distributor 1 2  Health facility 1 2  Drug shop 1 2  Other place (Specify) __________________________ 1 2 | | | |  |
| 406c | If you had a chance to select someone in your community to train as a local HIV self-test kit distributor, what qualities would you look for? (***Circle all that apply***) | Yes No  Someone who can keep secrets 1 2  Someone who can read and write 1 2  Someone who has ever tested for HIV 1 2  Someone who is approachable 1 2  Someone who is available at all times 1 2  Other quality (_____________________________) 1 2 | | | |  |
| 406d | Would you prefer peer-leader supervised or unsupervised HIV self-testing? | Peer-leader supervised HIV self-testing  Unsupervised HIV self-testing | Yes No DK/Not sure  1 2 98  1 2 98 | | |  |
| 406e | If you self-tested HIV-positive, would you be comfortable disclosing your HIV results to the following people? | Local HIV counsellor/Expert client  Your peer-leader  Spouse/sexual partner  Religious leader  Other HIV+ individuals in the community | Yes No DK/Not sure  1 2 98  1 2 98  1 2 98  1 2 98  1 2 98 | | |  |
| 407a | Individuals who will use the HIV self-test kits and test HIV-positive will be asked to go to a health facility to have their HIV results confirmed through blood-based rapid HIV testing. If you tested HIV-positive on the HIV self-test kit, would be willing to seek confirmatory HIV testing at an existing health facility? | Yes……………………………………………………….1  No………………………………………………………...2  Don’t know/not sure…………………………………….98 | | | | **IF 1, SKIP TO Q408** |
| 407b | If No or Don’t know/Not sure, please ask: Why would you not go for confirmatory HIV testing at an existing health facility after you have self-tested HIV-positive? | Yes No  Health facility is too far from where I live 1 2  I don’t like to go to the health facility 1 2  I don’t have money for transport to go there 1 2  Health workers are not good at keeping secrets 1 2  Other people may suspect that I am HIV-positive 1 2  Other reason (specify)_________________________ 1 2 | | | |  |
| 408 | If you were confirmed as being HIV-positive after undergoing blood-based rapid HIV testing at an existing health facility, would you be willing to start antiretroviral therapy? | Yes…………………………………………….………………….……….1  No……………………………………………………………………….....2  Don’t know/not sure………………………………………………….…..3 | | | | **IF 2 or 3, SKIP TO Q410** |
| 409a | If you were HIV-positive, how soon would you be willing to start HIV treatment? | Yes No  Immediately I am confirmed as HIV-positive 1 2  <1 week after my confirmed HIV-positive status 1 2  1 week after my confirmed HIV-positive status 1 2  2 weeks but <4 weeks 1 2  1 or more months since my confirmed HIV-positive status 1 2 | | | |  |
| 409b | If you were HIV-positive and were also willing to start antiretroviral therapy, where would you like to access your first dose of HIV treatment? | Yes No  Health facility 1 2  Home, delivered by local HIVST distributor 1 2  Home, delivered by a nurse from the health facility 1 2  Local HIV counsellor/Expert client in the community 1 2  Existing community ART group 1 2  From study team immediately after testing HIV-positive 1 2  Other place (specify)______________________________ 1 2 | | | |  |
| 410 | If you were HIV-positive but were not willing to start HIV treatment, what reasons would make you fail to initiate antiretroviral therapy after you have been confirmed as HIV-positive? | Yes No  Fear of beginning HIV treatment when I am still fine 1 2  Fear of antiretroviral drugs 1 2  Belief that HIV treatment drugs can worsen my situation 1 2  Fear of being able to adhere to the treatment as expected 1 2  Lack of someone to support me during HIV treatment 1 2  Not yet ready to start HIV treatment 1 2  Waiting to get weaker before I start treatment/still strong 1 2  Other reason (specify)______________________________ 1 2 | | | |  |

INTERVIEW ENDED AT: ____AM/PM:

**THANK YOU SO MUCH FOR YOUR TIME**

THE END
