# Supplementary material for: Feasibility and acceptability of a pilot, peer-led HIV self-testing intervention in a hyperendemic fishing community in rural Uganda
Source: PLoS One. 2020 Aug 7;15(8):e0236141. doi: 10.1371/journal.pone.0236141 (PMC7413506; doi:10.1371/journal.pone.0236141)
Supplement: S2 Study tool — (DOC) [file pone.0236141.s003.doc]

IMPLEMENTING A NETWORK-BASED, PEER-LED HIV SELF-TESTING INTERVENTION AMONG YOUNG PEOPLE AND ADULT MEN IN KASENSERO FISHING COMMUNITY, RAKAI DISTRICT

**BASELINE QUESTIONNAIRE – LUGANDA VERSION**

| **IDENTIFICATION OF THE PARTICIPANT** | |
| --- | --- |
| **INTERVIEWER CODE** | |  |  |  | | --- | --- | --- | |
| **INTERVIEW VENUE: __________________________________________________________**  **(One of two participating health facilities)** | |
| **STUDY COMMUNITY: __________________________________________________________** | |
| **PARTICIPANT NUMBER e.g. 01YM001 (01=community code, YM=young male, YF=young female, AM=adult male, AF= adult female, 001=participant’s number)** | |  |  |  |  |  |  |  | | --- | --- | --- | --- | --- | --- | --- | |
| **DATE** | |  |  |  | | --- | --- | --- |   **dd/mm/yyyy** |
| **SEX**  Male ……………………………………………………………………1  Female………………………………………………………………….2 | |  | | --- | |
| **FINAL DISPOSTION**  Interviewed ……………...........................………..……………….1  Refused………..............................................................................2  Other (specify_________________________________)…..….3 | |  | | --- | |

**INTRODUCTION**

| **Mr/Ms _____________________ (DO NOT WRITE THE NAME, JUST MENTION)**  Nga bwe maze okukweyanjuliira mu foomu gy’omaze okusayininga, amanya gange nze ____________________ era ndi wano ku lw’ettendekeero olw’ebyobulamu erya Makareere University School of Public Health**.** Nze omu ku tiimu eri mu kunonyereeza kunkola eyabantu okwekebela akawuka kasirimu bokanaboka**,** mu bavubuka ela n’abaami abakuze mumyaka mu Disitirikiti eno. Tul**i**basanyufu nnyo olw’okwetabamu kwo mu kunonyereeza kuno. Ebikunganyizibwa bino, bijja kuyamba gavumenti okuteekateeka, okutumbula ela nokubaga empereza yabantu okwekebela akawuka kasirimu bokanaboka eli abo ababeela kumyaalo. Okuddamu ebibuuzo kujja kutwaala nga essaawa emu. Buli kimu ky’onotuddamu kijja kukumiibwa mu kyaama era tebigenda kulagibwa muntu mulala yenna.  Kakano ntandike okubuuza ebibuuzo?  Respondent agrees to be interviewed…………………..1 respondent does not agree to be interviewed…………….2 |
| --- |

**INTERVIEW STARTED AT: ______AM/PM**

**100. GENERAL CHARACTERISTICS OF THE RESPONDENTS**

| **No.** | **QUESTION** | **CODING CATEGORIES** | **SKIP** |
| --- | --- | --- | --- |
|  | Waweza emyaka emekka egy’obukulu kumazalibwaago agakyasembyeyo? | AGE IN COMPLETED YEARS  DON’T KNOW…………….98 |  |
| 102 | Wali ogenzeko mu somero okusoma? | YES…………………………………….……….……………….…..1  NO…………………………………………..…………………..…...2 | **If 2 GOTO Q105** |
| 103 | Okyali mu somero?  (***Being in school here refers to attending formal classes (e.g. P1 or S1 )excluding vocational studies)*** | YES……………………………………………………………….…..1  NO…………………………………………………………………….2 | **If 1, GOTO Q105** |
| 104a | Wakoma kudaalaki ery’obuyigirize?  pulayimale, ssiniya, yunivasite oba tendekelo ddala? | PRIMARY (P.1 –P.7).………………………………………..……..2  PRIMARY PROFESSIONAL ………….………………..………...3  O’ LEVEL (S.1 – S.4) ….……….……………………….………….4  O’ LEVEL PROFESSIONAL ….……….………………..………..5  A’ LEVEL (S.5 – S.6) ……….……..…………………..…….….…6  UNIVERSITY………….……………………………….………..….7  OTHER TERTIARY (AFTER S.6) ……………….…………….…8  OTHER (Specify) ____________________________________9 |  |
| 104b | Wayisewo bangaki bukya ova mu somero? | LESS THAN A YEAR ………………………………………………1  1 YEAR AGO……………………………………..………………….2  2 YEARS AGO……………………………………..………………..3  3 YEARS AGO……………………………….…..………………….4  4+ MORE YEARS AGO…………………………………………….5  DON’T KNOW………………………………………………………..6 |  |
| 104c | Mbulila ensonga enkulu lwaki walekayo okusoma  (**Select all that apply**) | Yes No  LACK OF SCHOOL FEES, UNIFORM OR MATERIALS …….1 2  GOT MARRIED………………………………………………….....1 2  GOT SICK…………………………………………………………..1 2  NEEDED OR WANTED TO EARN MONEY…………………….1 2  NOT A GOOD STUDENT/FAILED IN SCHOOL……………......1 2  NOT INTERESTED IN SCHOOL…………………………………1 2  OTHER REASON ………………………………………………….1 2  (Specify ________________________________________________) | **Skip to Q105a** |
| 105 | Osoma kibiina ki?  (***Indicate actual class if below S6; otherwise, code 88 for University or 89 for other tertiary level***) | CLASS  UNIVERSITY …………………………………………….……………. 88  OTHER TERTIARY ……………………………………………………89 |  |
| 105a | Osobola okusoma ela nokuwandiika mululimilw’ oluzaalilanwa? | YES ……………………………………………………………………...1  NO …………………………………………………………………….….2 | **If 2, GOTO Q106** |
| 105b | **If yes, please read the following text**: “A*bantu abamu balina emputtu. Bw’omugamba okukola ekintu, akuddamu akudaala kumbe. Awo naawe n’ossalawo okumuleka.* ”  **Interviewer**: Has the participant been able to read the text? | YES, BUT WITH DIFFICULTY………………………………….….…...1  YES, WITH EASE (PROPER PRONOUNCIATION)…………….……2  NO, SHE WAS NOT ABLE TO READ THE TEXT……………………3 | **IF 3, ask Q105a again and code accordingly** |
| 106 | Obeera nani? | ALONE …………………………………………………………..1  FRIENDS…………………………………………………………2  MOTHER ALONE……………………………………………….3  FATHER ALONE………………………………………………...4  BOTH PARENTS………………………………………………...5  WITH OTHER RELATIVES……………………………………..6  HUSBAND/PARTNER ………………………………………….7 |  |
| 107a | Kati oli mufumbo?  (Obufumbo ntegeza obwempeta, okwanjula, obwomukooti, oba okubeela awamu nga abafumbo) | NEVER MARRIED………….…………………...........................1  IN RELATIONSHIP BUT NOT MARRIED….............................2  MARRIED/UNION…………………………………………..……3  DIVORCED/SEPARATED…………….……..............................4  WIDOWED…………………..……………..….............................5 | **IF 1,4,5 GOTO Q108**  **IF 2, skip to Q107c** |
| 107b | Oli mubufumbo bwangeri ki? | MONOGAMOUS……………………………….……………...…1  POLYGAMOUS……….………………………...………………..2 |  |
| 107c | Omwagalwawo alina emyaka emeka?  **INTERVIEWER NOTE:** ***If not married, ask age for most recent sexual partner*** | AGE IN YEARS  DON’T KNOW 98  Not Applicable 97 |  |
| 108 | Osoma diini ki? | CATHOLIC…………………………………………....….........………...1  ANGLICAN/PROTESTANT……………….……...............……………2  MOSLEM………………………………………………...............………3  PENTECOSTAL /BORN AGAIN / EVANGELICAL…........................4  SEVENTH DAY ADVENTIST……….………………………................5  ORTHODOX…………………………………………………………..…6  OTHERS(specify)………………………………………......……...........7 ________________________________________ |  |
| 109 | Mulimuki omukulu gwokola ela gwosinga okumalilako obuddebwo obusinga obungi? | FISHING……………………………………………………………….…1  FISHING-RELATED ACTIVITY (E.G. NET REPAIRING)…………..2  PEASANT FARMER…………………..………..……....……………...3  SALARIED…………………………………………..…….……………..4  BUSINESS/COMMERCIAL……………………..………………….….5  CASUAL WORKER………………………..…………………………...6  HOUSE WIFE…………………...........………….………………….…..7  PUPIL/STUDENT/NO OCCUPATION....…………………………….8  OTHER (SPECIFY)………………………………..…………..….……9 ______________________________________________ | **IF7,8 GOTO Q111** |
| 110 | Okugerageranya, osasaanya sente meka omwezi? | LESS THAN 10,000/=………………..…….………………………..…1  10,000-30,000/=………………………………………………………...2  31,000-50,000/=………………..………………………..…….......…...3  51,000-100,000………………………….……..….………………...….4  101,000-500,000……………………………...……………….…..…...5  >500,000……………………………………………………………...…6 |  |
| 111 | Wali okwatidwa ko kundwadde y’ekikaba? | YES……………………………………………………………………...1  NO…………………………………………………………………....….2 | **IF 2, GOTO**  **Q115** |
| 112 | Wanonya obujjanjabi kundwadde y’obukaba? | YES…………………………………………………………….………..1  NO……………………………………………………………………….2 |  |
| 113 | Oluvanyuma lwokutegera nti olina endwadde yobukaba watwala bangaki olyoke ononye obujanjabi? | SAME DAY ……………………………………………………………..1  1-2 DAYS ……………………………………………..………………..2  3-5 DAYS……………………………………………….………………3  1 WEEK………………………………………………….……………..4  MORE THAN 1 WEEK…………………………………….………….5 |  |
| 114 | Wanonyawa obujanjabi? | SHOP………………………………………………….……….……….1  PHARMACY………………………………………………….….....…..2  GOVT. HOSPITAL/HEALTH CENTRE/CLINIC……………..……..3  PRIVATE DOCTOR/NURSE/CLINIC……….………...…………….4  HERBAL/ TRADITIONAL PROVIDER …………………………… ..5  OTHER, **SPECIFY**: ____________________________________6 |  |
| 115 | Olina esimu eyomungalo? | YES………………………………………………………………...1  NO………………………………………………………………….2 | **IF 2, GOTO Q201** |
| 116 | Olina simu kikaaki? | ORDINARY PHONE………………………………………………1  SMART PHONE WITH TOUCH SCREEN……………………..2  OTHER TYPE (SPECIFY) ___________________________3 |  |

**200. SEXUAL BEHAVIOUR**

.

Kakati ngenda ku kubuuzayo ebibuuzo ebikwaata ku kwegatta mu mukwaano era ebibuuzo ebimu bigenda kuba bikwaata ku neyisayo etamanyiddwa balala. Ebimu ku bibuuzo bino byamunda ddala. Kkyoka ng’okunonyereeza kuno bwe kuli okwekyaama, erinnya lyo terigenda kuwandikiibwa ku lupapula luno, teri n’omu agenda kumanya oba okwataganya by’onoddamu naawe. Tunasanyukira nnyo okwetabamu ng’oddamu ebibuuzo mu bwesimbu nga bwe kisoboka**.**

| No. | QUESTION | CODING CATEGORIES | SKIP |
| --- | --- | --- | --- |
| 201 | Wali wegaaseko mu mukwano ? | YES ……………………………………....1  NO ………………………………….….....2 | **IF 2,**  **GOTO Q301** |
| 202 | Walina emyaka emeka egy’obukulu lwe wasokeera ddaala okwegatta mu mukwano ? | AGE IN YEARS ……………….  DON’T KNOW…..………….. 98 |  |
| 203 | Omuntu gwewasoka okwegatta naye mu mukwano wali omuyita otya | Boyfriend/girlfriend….………………………….1  Husband/wife….………………………………...2  Stranger………………………………………….3  Brother/sister……………………………………4  Teacher…………………………………….….....5  Uncle/Auntie……………………………………..6  Father.……………………………………………7  Sex worker…………………………….…………8  Fish monger/fish trader ………………………..9  Other relative (specify)_________________..10  Other (Specify) _____________________.....11 |  |
| 204 | Omuntu gwewasoka okwegatta naye yalina emyaka emekka? | Same age as me ……………………………….1  Younger than me ……………………………….2  1-2 years older than me………………………..3  3-4 years older than me………………………..4  5 or more years older than me………………..5  Don’t know………………………………………98 |  |
| 205 | Omulundi gwewasooka okwegatta, wakikola lwakuba nti wali onywedde kumwenge oba ebiragalalagala? | YES …………………………………………… …1  NO…………………………………………………2  Don’t know/Don’t Remember………………….98 |  |
| 206 | Ddi lwokyasembyeeyo okwegatta mu mukwano? | Within 1 week………………………..……..…….1  Within 1 month………………………….……..….2  >1 but <3 months …………………….……..…...3  >3 months ago………..……..……………….…...4 | **IF 4**  **GOTO Q213** |
| 207 | Omuwendo gw’abantu abagalana be begatta nabo mu mukwaano gwawukana okuva ku muntu omu okutuuka ku mulala. Abantu abamu batera okwegatta n’omuntu omu, abalala babbiri oba okusingawo.  Mu myezi 3 egyiyise, abantu bameka bewegasse nabo mu mukwaano? | _____________Partners |____|____|  DON’T KNOW 98  DECLINED TO ANSWER 99 |  |
| 208 | Mu myeezi 3 egyiyise, ddi lw’obadde okozesa kkondomu nga wegasse mu mukwaano n’abagalwaabo / baganzi bo bonna bano? | Always…………………………………………...1  Sometimes ……………………………………...2  Rarely…………………………………………….3  Never …………………………………………….4 |  |
| 209 | Omuntu gw’okyasembyeeyo okwegatta naye mu mukwano omuyita otya? | Boyfriend/girlfriend….………………………….1  Husband/wife….………………………………...2  Stranger………………………………………….3  Brother/sister……………………………………4  Teacher…………………………………….….....5  Uncle/Auntie……………………………………..6  Father..……………………………………………7  Sex worker…………………………….…………8  Fish monger/fish trader ………………………..9  Other relative (specify)_________________..10  Other (Specify) _____________________.....11 |  |
| 210 | Omuntu oyo gw’okyasembyeeyo okwegatta naye yalina emyaka emekka? | Same age as me ………………………………. 1  Younger than me ………………………………. 2  1-2 years older than me ………………………..3  3-4 years older than me ………………………..4  5 or more years older than me ………………..5  Don’t know ……………………………………. 98 |  |
| 211 | Lowooza **KU MULUNDI GW’OKYASEMBYEYO** okwegatta mu mukwano ne muganziwo ono, mwakozesa kkondomu? | YES ……………………….…………………….1  NO …………………………….………………...2 | **IF 2 GOTO Q213** |
| 212 | Lowooza ku mirundu gyonna gye wegasse mu mukwaano ne muganziwo ono **MU MYEEZI 3 EGYIYISE,** osobola okugamba nti wakozesa kondomu buli kiseera, ebiseera ebimu, oba tewakozesakowadde? | Never……………………………..……………...1  Sometimes ………………….……..….....….…..2  Always …………………..……………………....3  NA (Had no sex in last 12 months)…..…4  Don’t know/don’t remember ……….………....98 |  |
| 213 | Abantu abamu berarikirira okufuna endwadde zobukaba. Gwe obweralikirivu bwo bulibutya oba bwali butya okufuna endwadde zekikaba okuva kumwagalwawo bwe mwegatta mumyezi 3 egiyise? | Very concerned …………………………………1  Somewhat concerned ………………………….2  Not really concerned …………………………...3  Not at all concerned ……………………………4  Don’t know ……………………………………..98 |  |
| 214 | Mu kiseera kino mukozesa kkondomu? | YES ………………………………………….….1  NO ………………………………………….…...2 | **IF 2 GOTO Q217** |
| 215 | Obanga mukiseera kino mukozesa kkondomu, lwaki muzikozesa? | Family planning……………….…………….…..1  HIV/other STI prevention………………...…….2  Both ……………………….……………………..3 |  |
| 216 | Mukozesa kkondomu z’abaami oba kkondomu z’abakyaala? | Male condom……………….…………....……...1  Female condom……….…….…...……..............2  Both…………………………...………………....3 |  |
| 217 | Omanyiyo ekifo omuntu gyayinza okufuna kkondomu? | YES ……………………………………………….1  NO ………………………………………………...2  DON'T KNOW .………………………………….98  REFUSED TO ANSWER ..…………………….99 |  |
| 218 | Singa oba nga oyagadde, osobola okwefunira kkondomu? | YES . . . . . . . . . . . . . . . . . . . . . . . . . …… . . 1  NO . . . . . . . . . . . . . . . . . . . . . . . . . …… . . 2  DON'T KNOW/UNSURE . . . . . . . . . . …... .. 98 | **IF 2, 98 skip to**  **Q220** |
| 219 | Bwoba nga tosobola kwefunila kkondomu nsongaki ekugaana? | I fear to ask for a condom …………………….…..1  I cannot afford the cost of condoms ……….…….2  I don’t know where to find them …………..……..3  Place is very far …………………………………...4  My partner does not like condoms ………………5  My religion does not allow me to use condoms……6  Other (specify _____________________)……..7 | **IF 3, please check response to Q 217zx** |
| 220 | Mu myeezi 3 egyiyise, mirundi emekka gyosose okunywa omwenge nga tonaba kwegatta nomwagalwawo akyasembyeyo? | Never/have no partner ………………………..1  Some of the time………………………………2  Most of the time……………………………….3  Always………………………………………….4 |  |
| 221 | Oluusi n’oluusi abantu abamu, batera okwegatta mukwaano olw’okufuna ebintu, ensimbi, oba obuyaambi.  Mu myeezi 3 egyiyise, wegaseeko mu mukwaano olw’ebintu, ensimbi/sente oba obuyambi bye wafuna okuva kumwagalwawo yenna? | YES ………………………………..………….1  NO ……………………………………..……...2  DON'T KNOW .……………………………….98  REFUSED TO ANSWER ..………………….99 |  |
| 222 | Obw’olumu oba bulijjo abakyaala abamu batera okuwaayo ebintu, ensimbi/sente oba obuyambi olw’okwegatta mu mukwaano.  Mu myeezi 3 egyiyise, owaddeyo ebintu, ensimbi / sente, oba obuyambi eli omwagalwawo yenna olw’okwegatta mu mukwaano? | YES ……………………………………..…….1  NO ……………………………………..……...2  DON'T KNOW .……………………………….98  REFUSED TO ANSWER ..………………….99 |  |

**300. HIV/AIDS – GENERAL KNOWLEDGE, COMPREHENSIVE KNOWLEDGE, HIV TESTING**

**(KUMANYA OKUTANDIKIRWAKO N’OKUGENDA EBUZIBA KU KAWUKA KASIRIMU NOKUKEEKEBEZA)**

| **KNOWLEDGE OF HIV/AIDS (OKUMANYA KU KAWUKA KASIRIMU)** | | | |
| --- | --- | --- | --- |
| 301 | Wali owulidde kundwadde yamukenenya eletebwa akawuka ka sirimu? | Yes……………………………….…….1  No ……………………………………..2 | **IF 2, GOTO Q331** |
| 302 | Singa omusajja oba omukazi alina akawuka kasirimu, kyatteeka nti omwagalwawe naye akalina? | Yes…………………………………….1  No ……………………………………..2  DON’T KNOW ……………………….98 |  |
| 303 | Kisoboka omuntu alabika obulungi okuba nga alina akawuka kasirimu? | Yes…………………………………….1  No ……………………………………..2  DON’T KNOW ……………………….98 |  |
| 304 | Singa maama abanga alina akawuka kasirimu asobola okukasiiga omwanawe alimulubuto? (Unprompted) | Yes…………………………………….1  No ……………………………………..2  DON’T KNOW ……………………….98 |  |
| 305 | Omanyi ekifo omuntu gyayinza okwekebeza akawuka kasirimu? | Yes………………………….………….1  No ……………………….……………..2 |  |
| 306 | Abantu basobola okukendeeza emikisa egyokufuna akawuka ka sirimu nga begatta n’omuntu omu atalina sirimu ela nga teyegatta namuntu mulala yenna? | YES . . . . . . . . . . . . . . . . . . . . . . . . .1  NO . . . . . . . . . . . . . . . . . . . . . . …. 2  DON'T KNOW . . . . . . . . . . . . . .. 98 |  |
| 307 | Abantu basobola okufuna akawuka kasirimu nga bakafuna okuva kunsiri okubaluma? | YES . . . . . . . . . . . . . . . . . . . . . . . . .1  NO . . . . . . . . . . . . . . . . . . . . . . …. 2  DON'T KNOW . . . . . . . . . . . . . .. 98 |  |
| 308 | Abantu basobola okukendeza emikisa egyokufuna akawuka kasirimu nga bakozesa kkondomu buli lwebegatta? | YES . . . . . . . . . . . . . . . . . . . . . . . . .1  NO . . . . . . . . . . . . . . . . . . . . . . …. 2  DON'T KNOW . . . . . . . . . . . . . .. 98 |  |
| 309 | Abantu basobola okufuna akawuka kasirimu nga bayita mukuliila awamu emere nabo abalin akawuka kasirimu? | YES . . . . . . . . . . . . . . . . . . . . . . . . .1  NO . . . . . . . . . . . . . . . . . . . . . . …. 2  DON'T KNOW . . . . . . . . . . . . . .. 98 |  |
| 310 | Abantu basobola okufuna akawuka kasirimu olwakuba nti babaloze? | YES . . . . . . . . . . . . . . . . . . . . . . . . .1  NO . . . . . . . . . . . . . . . . . . . . . . …. 2  DON'T KNOW . . . . . . . . . . . . . .. 98 |  |
| 311 | Akawuka kasirimu kasobola okuva kumaama nekakwata omwana:  Nga maama ali,  Lubuto?  Mukiseera ekyokuzaala?  Mukiseera ekyokuyonsa? | YES NO DK DURING PREG ……. ….. 1 2 98  DURING DELIVERY…. . .1 2 98  BREASTFEEDING … . 1 2 98 |  |
| 312 | Omuntu asobola okwekuuma okufuna akawuka kasirimu nga ayita mukwekuuma obutegatta? | YES . . . . . . . . . . . . . . . . . . . . . . . . .1  NO . . . . . . . . . . . . . . . . . . . . . . …. 2  DON'T KNOW . . . . . . . . . . . . . .. 98 |  |
| 313 | Okukomolebwa kwabasajja kusobola okuyambako okukendeeza emikisa gyokukukwatibwa kw’akawuka kasirimu? | YES . . . . . . . . . . . . . . . . . . . . . . . . .1  NO . . . . . . . . . . . . . . . . . . . . . . …. 2  DON'T KNOW . . . . . . . . . . . . . .. 98 |  |
| 314 | Waliyo edagala dokita oba nansi lyayinza okuwa omukyala ali olubuto okutaasa omwana obutakwatibwa kawuka kasirimu? | YES . . . . . . . . . . . . . . . . . . . . . . . . .1  NO . . . . . . . . . . . . . . . . . . . . . . …. 2  DON'T KNOW . . . . . . . . . . . . . .. 98 |  |
| **ACCEPTING ATTITUDES ABOUT PEOPLE LIVING WITH HIV** | | | |
| 315 | Osobola okugula enva kumuntu singa omanya nti alina akawuka kasirimu? | YES . . . . . . . . . . . . . . . . . . . . . . . . .1  NO . . . . . . . . . . . . . . . . . . . . . . …. 2  DON'T KNOW . . . . . . . . . . . . . .. 98 |  |
| 316 | Singa omu kubomumakago akwatibwa akawuka kasirimu wandyagadde kisigale nga kyama oba nedda? | YES, REMAIN A SECRET………… 1  NO . . . . . . . . . . . . . . . . .. . . . . . 2  DK/NOT SURE/DEPENDS ..……… 98 |  |
| 317 | Singa omukubo’mumakago akwatibwa akawuka kasirimu wandyagadde okumujanjabira mumakago? | YES . . . . . . . . . . . . . . . . . . . . . . . . 1  NO . . . . . . . . . . . . . . . . . . . . . . . . . 2  DK/NOT SURE/DEPENDS ……….. 98 |  |
| 318 | Mundowoozayo, singa omusomesa omukyala abera nga alina akawuka kasirimu naye nga simulwadde, asigale nga akirizibwa okusomesa musomero? | SHOULD BE ALLOW ……………. 1  SHOULD NOT BE ALLOWED …… 2  DK/NOT SURE/DEPENDS ……… 98 |  |
| **HIV TESTING (OKWEKEBEZA AKAWUKA KASIRIMU)** | | | |
| 319 | Wali wekebeza kukawuka kasirimu? | Yes…………………….……………….1  No ………………………….…………..2 | **IF 2**  **GOTO**  **Q328** |
| 320 | Wakekebeza emirundi emeka akawuka kasirimu? | Number ________ |  |
| 321 | Ddi lwewasemba okwekebeza akawuka kasirimu? | Less than 1 month ago ………….………….1  1-3 months ago ………………………………2  More than 3 months ago ……………………5  Can’t remember ………………………...……6 |  |
| 322 | Osobola okumbulilako kubyava mukukeberebwa akawuka kasirimu okukyasembyeyo? | Yes………………………………………..….1  No ……………………………………………2 | **IF 2**  **GOTO**  **Q324** |
| 323 | Bwoba osobola okumbulilako, ebyavamukeberebwa byali bitya? | Positive…………………………….………1  negative ………………………….………..2  indeterminate……………………..………3  did not receive results ……………..…4 |  |
| 324 | Wali obuliddeko omwagalwawo bwoyimiridde kukawuka kasirimu? | Yes……………………………….…….….1  No ………………………………………....2  No partner/not applicable……..…….3 |  |
| 325 | **Interviewer: Skip to Q328 if respondent was NOT HIV-positive at last test.**  Bwoba nga olina akawuka kasirimu, olina wewewandiisa okufuna endabirira kukawuka kasirimu? | Yes ………………………………..……1  No ……………………………..………..2 | **IF 2, SKIP TO Q328** |
| 326 | Oli kudagala lya ARVs kati? | Yes…………………………….1  No ……………………………..2 | **IF 2 GOTO**  **Q328** |
| 327 | Watandikaddi okumila ARVs okujanjaba akawuka kasirimu? | ______________Months  _______________Years |  |
| 328 | Wali obuzizako omwagalwawo gwolina kati oba nga yali yekebeza kukawuka kasirimu? | Yes……………………………….……….…….1  No ……………………………………….….…..2  HAVE NO PARTNER/NOT APPLICABLE.....3 | **IF 3, SKIP TO Q331** |
| 329 | Omanyi oba omwagalwawo gwolina kati yali yekebeza kukawuka kasirimu? | Yes…………………………………………….1  No ……………………………………………..2 |  |
| 330 | Mwali mwekebezako akawuka kasirimu mwembi nomwagalwawo nga abaagalana? | Yes…………………………………………….1  No ……………………………………………..2 |  |
| 331 | **Kati njagala kukubuuzako ebibuuzo ebikwata kubwobulamubwo.** . Mu myeezi 3 egiyise wafunako obulwadde nga wabufuna mukwegatta? | YES . . . . . . . . . . . . . . . . . . . . . . . . .1  NO . . . . . . . . . . . . . . . . . . . . . . …. 2  DON'T KNOW . . . . . . . . . . . . . .. 98 | **CROSS-CHECK WITH Q111** |
| 332 | Omulundi gwewasembayo okufuna (obuzibu okuva 331)wafunako okuwabulwa oba okunonya obujanjabi? | YES . . . . .. . . . . . . . . . . . . . 1  NO . . . . . . . . . . . . .. . . . …2 | **IF 2 GOTO**  **Q401** |
| 333 | Wagendawa?  Waliwo awalala wewalaga?  PROBE TO IDENTIFY EACH TYPE OF SOURCE.  IF UNABLE TO DETERMINE IF PUBLIC OR PRIVATE SECTOR, WRITE THE NAME OF THE PLACE.  (NAME OF PLACE(S) | **PUBLIC SECTOR**  GOVERNMENT HOSPITAL . . . . . . . .1  GOVT. HEALTH CENTER . . . . . . . .2  STAND-ALONE VCT CENTER . . . .3  FAMILY PLANNING CLINIC . . . . . 4  OUT REACH . . . . . . . . . . . . . . 5  VILLAGE HEALTH TEAM ………… 6  OTHER PUBLIC____ ______7 (SPECIFY)  **PRIVATE MEDICAL SECTOR**  PRIVATE HOSPITAL/CLINIC ……… 8  STAND-ALONE VCT CENTER …… 9  PHARMACY/DRUG SHOP . … 10  PRIVATE DOCTOR/NURSE/  MIDWIFE …………………. 11  OUT REACH . . . . . . . . . . . . . . . 12  TASO . . . . . . . . . . . . . . . . . . 13  AIDS INFORMATION CENTRE .…. 14  OTHER PRIVATE/NGO/MEDICAL _____________________________ 15  (SPECIFY)  OTHER ______________________ 16  (SPECIFY) |  |

**400 KNOWLEDGE, ATTITUDES AND PERCEPTIONS TOWARDS HIV SELF-TESTING**

**(OKUMANYA, ENDOWOOZA NEBISUBIRWA KUNKOLA EYO KWEKEBELA AKAWUKA KASIRIMU)**

| 401 | Wali owulidde kunkola ey’okwekebela akawuka kasirimu nga okozesa ka kit kebayisa mukamwa? | Yes…………………………………………..1  No……………………………………………2  Don’t know/not sure……………………….98 | | | **If 2 , 98 SKIP TO preamble before**  **Q 403a** |
| --- | --- | --- | --- | --- | --- |
| 402 | Bwekiba bwekityo, ki kyewawulila kunkola eno? | HIV self-testing can be done outside formal health facilities  HIV self-testing does not use blood to detect HIV  HIV self-testing uses “saliva” to detect HIV  HIV self-testing is easy to perform  HIV self-testing yields accurate results | | Yes No DK  1 2 98  1 2 98  1 2 98  1 2 98  1 2 98 |  |
|  | **INTERVIEWER: PLEASE LEVEL THE PARTICIPANT’S UNDERSTANDING OF HIV SELF-TESTING.**  **HIV self-testing is a procedure in which an individual collects an oral swab (from the mouth) using a kit (i.e. HIV self-test kit), places the kit in a testing vial (bottle) and waits for 20 minutes to read and interpret the HIV test results. It works more or less in the same as a pregnancy test kit. HIV-positive results are shown with two red lines on the screen of the kit while HIV-negative results are shown with one line. If no lines show on the screen, the test is considered to be invalid.** | | | |  |
| 403a | Wandyagadde okwekebera akawuka kasirimu wekanawekka singa ebikozesebwa bikutuusibwako ela nga byabwelele? | Yes………………………………………………….………..1  No…………………………………………………..…………2  Don’t know/not sure…………………………….………….98 | | |  |
| 403b | Singa ebikozesebwa mukwekebela akawuka kasirimu bibabize wandiagadde kubijjawa? | Yes No DK  Hospital/health center/clinic 1 2 98  Pharmacy 1 2 98  Drug shop 1 2 98  Community-based distributor’s home 1 2 98  Church/mosque 1 2 98  Other place (Specify_________________) 1 2 98 | | |  |
| 403c | Singa ebikozesebwa okwekebela akawuka kasirimu biba biletedwa gyoli wandyagadde kuyambibwa mungeliki okusobo okwekebela bulungi ngatokoze nsobi yonna? | Yes No  How to obtain the oral swab ……………………………1 2  How to perform the test itself……………………………1 2  How to read the results…………………………………..1 2  How to interpret the results………………………………1 2  Pre- and post-testing counselling……………………….1 2  Referral for HIV care if HIV-positive…………………….1 2  How to dispose of the kit after use……………………...1 2  Other support (Specify ____________________)…….1 2 | | |  |
| 404 | Singa ebikozesebwa mukwekebela akawuka kasirimu biba byakugabibwa kubwelele mukitundukyo ani gwewandyagadde abigabe? | Yes No DK  Trained community health volunteer 1 2 98  Friend/relative 1 2 98  Partner (spouse/boyfriend/girlfriend) 1 2 98  Local council official 1 2 98  Religious official 1 2 98  Other (specify___________________) 1 2 98 | | |  |
| 405 | Laga oba nga:   1. Tokiliziganyiza ddala, 2. Tokiliza era towakanya 3. Okililiza ddala   ebyo ebiwandikidwa kumukono gwo ogwa ddyo. | **1=Strongly disagree 2=Neither agree nor disagree 3=Strongly agree** | Yes No Don’t know | |  |
|  |  | Singa kits zokwekebera akawuka akasilimu zibaawo nja kuba musanyufu okufunayo nenjikozesa  .  Bu kits bwokwekebela akawuka kasilimu buja kwongera obwenzi mukitundukino.  Abantu bokumyalo tebagala kwekebeza kawuka ka silimu.  Abantu bokumyalo tebaja kwagala a kozesa kits ezikebela Akawuka kasilimu nebwozibawera obwerere.  Bu kits obwokwekebela akawuka kasilimu bujjakwongera omuwendo gwabantu abamanyi bwebayimilide kukawuka kasilimu.  Singa nfuna okutendekebwa okumala mpulira nekakasa nga nsobola okozesa bulungi ka kit okwekebela akawuka kasilimu.  Abantu bajja kulowooza nti abo abakozesa bu kit bwokwekebela akawuka ka silimu benzi  Silowooza nti ebiva mukwekebela akawuka kasilimu nti biba bitufu.  Nandiyagadde okozesa ekola eziliwo ezokwekebela akawuka kasilimu mukifo kya kit.  Abantu abasing mukitundu kino baja kwetanila enkozesa yabu kit obwokwekebela akawuka kasilimu. | 1 2 3 98  1 2 3 98  1 2 3 98  1 2 3 98  1 2 3 98  1 2 3 98  1 2 3 98  1 2 3 98  1 2 3 98  1 2 3 98 | |  |
|  | **INTERVIEWER: PLEASE INTRODUCE THE PROPOSED HIV SELF-TESTING STUDY HERE.**  **Tulina enteekateeka eyokola okunonyeleza ela nekigendererwa ekyokugaba ebikozesebwa mukwekebela akawuka kasirimu eli abo abalondedwe mukitudu. Okugaba ebikozesebwa bino kujja kolebwa abo abanaaba batendekebwa elanga balondedwa okukola omulimu ogwo mukitundukyo. Abo abanaaba bavunanyizibwa kukutuusa ebikozesebwa bino mubantu ela bajja kusomesa abantu enkozesa yabyo ela no kubabulila engeli gyebataputamu ebyo ebiba buvudde mukukebela akawuka kasirimu.** | | | |  |
| 406a | Wandiyagadde okufuna ebikozesebwa okuva eli oyo atendekedwa mukitundu kyo? | Yes……………………………………………………….1  No………………………………………………………...2  Don’t know/not sure…………………………………….3 | | | **IF 2,3 – SKIP TO Q407** |
| 406b | Byekiba byekityo wangyagadde ekivulu ky’okugaba ebikozesebwa mukwekebela akawuka kasirimu kibeewo? | Yes No  Own home 1 2  Own work-place 1 2  Local distributor’s work place 1 2  Anywhere within the community but not at home 1 2  Home of local HIV self-test kit distributor 1 2  Health facility 1 2  Drug shop 1 2  Other place (Specify: ______________________) 1 2 | | |  |
| 406c | Singa obadde nomukisa okulonda omuntu owokutendekebwa okubeela oyo asasaanya ebikozesebwa mukwekebeza akawuka kasirimu, bisanyizoki byewanditunulidde? | Yes No  Someone who can keep secrets 1 2  Someone who can read and write 1 2  Someone who has ever tested for HIV 1 2  Someone who is approachable 1 2  Someone who is available at all times 1 2  Other quality (_________________________) 1 2 | | |  |
| 406d | Wandiyagadde okwekebela akawuka akaleta silimu nga oyambibyako omuntu eyatendekebwa mukitundukyo oba nedda? | Yes No DK/Not sure Peer-leader supervised HIV self-testing 1 2 98  Unsupervised HIV self-testing 1 2 98 | | |  |
| 406e | Singa wekebela newesanga nga olina akawuka akaleeta silimu waliwulidde emilembe okubulilako abantu bano wamanga kubivude mukwekebeza akawuka nkasilimu? | Yes No DK/Not sure  Local HIV counsellor/Expert client 1 2 98  Your peer-leader 1 2 98  Spouse/sexual partner 1 2 98  Religious leader 1 2 98  Other HIV+ individuals in the community 1 2 98 | | |  |
| 407a | Abantu abanekebela nebasanga nga balina akawuka kasirimu balina okweyongelayo mu ddwaliro nebongera okeberebwa omusayi okukakasa ebyo bebaazudde.  Singa obadde wekebedde nozuula nti olina akawuka kasirimu wandyagadde okweyongelayo muddwalilo okukakasa obanga ddala okalina? | Yes……………………………………………………….1  No………………………………………………………...2  Don’t know/not sure…………………………………….3 | | | **IF 1, SKIP TO Q 408** |
| 407b | Lwaki tewandiyagadde kugenda ku dwaliiro lya government okudamu okwekebeza akawuka kasilimu okukakasa ebivudde mumusayi. | Yes No  Health facility is too far from where I live 1 2  I don’t like to go to the health facility 1 2  I don’t have money for transport to go there 1 2  Health workers are not good at keeping secrets 1 2  Other people may suspect that I am HIV-positive 1 2  Other reason (specify)_________________________1 2 | | |  |
| 408 | Singa obadde wekebedde nokizuula nti olina akawuka kasilimu ela nekikakasibwa nemudwaliro nga omusayi gwo gukebeddwa, wandyagadde otandikilawo okumila empeke za ARVs amanguddala? | Yes……………………………………………………….1  No………………………………………………………...2  Don’t know/not sure…………………………………….3 | | | **IF 2 OR 3 SKIP TO Q410** |
| 409 a | Singa wesanga nga olina akawuka akaleta silimu wandiyagade kutandika di edagala eliweweeza akawuka kasilimu | Yes No  Immediately I am confirmed as HIV-positive 1 2  <1 week after my confirmed HIV-positive status 1 2  1 week after my confirmed HIV-positive status 1 2  2 weeks but <4 weeks 1 2  1 or more months since my confirmed HIV-positive status 1 2 | | |  |
| 409 b | Singa obadde olina akawuka kasilimu ela nga oyagala okutandika obujanjabi wandyagadde kufunilawa dose yo esoka eyedagala eliweweeza akawuka ka silimu. | Yes No  Health facility 1 2  Home, delivered by local HIVST distributor 1 2  Home, delivered by a nurse from the health facility 1 2  Local HIV counsellor/Expert client in the community 1 2  Existing community ART group 1 2  From study team immediately after testing HIV-positive 1 2  Other place (specify)______________________________ 1 2 | | | **END AFTER THIS QUESTION** |
| 410 | Singa obadde oyina akawuka kasilimu naye nga toyagala kutandika dagala eliweweza akawuka kasilimu, nsongaki ezalikulemeseza okutandika edagala ngate omaze okukakasibwa nti olina akawuka akaleta silimu. | Yes No  Fear of beginning HIV treatment when I am still fine 1 2  Fear of antiretroviral drugs 1 2  Belief that HIV treatment drugs can worsen my situation 1 2  Fear of being able to adhere to the treatment as expected 1 2  Lack of someone to support me during HIV treatment 1 2  Not yet ready to start HIV treatment 1 2  Waiting to getting weaker to start treatment/still strong 1 2  Other reason (specify)______________________________ 1 2 | | |  |

INTERVIEW ENDED AT: ____AM/PM:

**THANK YOU SO MUCH FOR YOUR TIME**

THE END
