# Supplementary material for: Feasibility and acceptability of a pilot, peer-led HIV self-testing intervention in a hyperendemic fishing community in rural Uganda
Source: PLoS One. 2020 Aug 7;15(8):e0236141. doi: 10.1371/journal.pone.0236141 (PMC7413506; doi:10.1371/journal.pone.0236141)
Supplement: S4 Study tool — (DOC) [file pone.0236141.s005.doc]

**FOCUS GROUP DISCUSSION GUIDE**

**[QUALFORM – FGD/HIVST/01]**

**Study Title: Implementing a Network-based, Peer-led HIV Self-testing Intervention to Improve HIV Testing and Linkage to HIV Care among Young People and Adult Men in Kasensero Fishing Community, Rakai District**

**FOR OFFICIAL USE ONLY**

Date of Interview: ______/ _______/ ____________

Community Name: _______________________________

Venue: ______________________________

Language of interview: _______________________

Number of participants: _______________________

Time started: ________________ Time ended: _________________

**FGD identifier: ______________________________**

**Note: The FGD identifier** should be composed of the abbreviation “FGD” followed by the category of participants engaged in the FGD- abbreviated as YM for FGD for young men; YF – for FGD for young females; AM – for FGD for adult men; community code (KYE for Kyebe; GWA for Gwanda and KAS for Kasensero); date of interview in the format ***yy/mm/dd*** and FGD number (3 digits) assigned cumulatively. For example, if the first FGD was done among young men in Kyebe on May 15^th^, 2019; this FGD’s identifier should be in the form: **FGD/YM/KYE/19/05/15/001.**

**TEAM INTRODUCTION**

(Mwebale okukiriza okwetaba mukunonyereza kuno okululubirira o**kutandikawo enkola ey’okwekebela akawuka kasirimu ng’ezimbiddwa kumusingi ogw’abantu okkolela awamu ela nga bakulemberwa banaabwe okusobola okutumbula okwekebeza akawuka kasirimu ela nokuyungibwa kubujanjabi bwa kawuka asirimu eri abavubuka wamu nabasajja abakuze mumyaka ab’omukitundu ky’ekasensero kumwalo mu Rakai District.** Kati tweteeseteese bulungi okutandika okukubaganya ebirowoozo. Tugende maaso nokweyanjula tusobole okumanyagana mukukubaganyaebirowoozo kuno. Nze …………………………………….. era ndinamwe leero okulaba nti nkubiriza okukubaganya ebirowoozo kuno. Munange ye…………………………………era aja kuba nga awandiika byetunateesako. Nga bwenjogede, Emboozi yaffe yonna egenda kukwatibwa kukatambi. Okukuuma ebyama ebyabuliomu, tujja kukozesako lina lyadini lyoka. Byonoyogera tebijja kukulaga gwe nga omuntu, wulira emirembe okutubulira bulikimu kyolowooza. Katutandikire obuuyi buno (akubiriza olukiiko asonge kudyo). Tukusaba otubulile erinyalyo, wa gyova, naki kyosubira okuva mukukubaganya ebirowoozo kuno. Tujja kutambula nga twetoloola bwetuti (akubiriza aja kulaga) paka bwetunagwayo).

**GROUND RULES**

(Nga tetunaba kutandika, katwejukanye amateeka gano wamanga buli omukuffe gateekedwa okusamu ekitiibwa okusobola okubeera nokukubaganya ebilowoozo okulungi)

1. Tukubiriza buli omu wano okubako nekyakesa, naye ekisinga obukulu, kwekugoberera omulamwa gwetuteesako. Tujja kuba basnyufu singa tuleka muntu omu nayogera awatali kumutataganya. Tujja kubajukiza buli omu okukuuma ebyamune; osabibwa obutabulira muntu yena atabadde mukutesa kuno buli omu kyateseza wano.
2. Mukuteesa kwaffe leero, tukusaba okukiteka mundowooza nti twetaga ndowoozayo. Twagala kumanya byolowooza, biki byolowooza abantu abalala byebalowooza, nabiki byomanyi abantu abalala byebayisemu. Ekigendererwa kyokuteesa kuno sikwogera kubiki byoyisemu nga omuntu. Naye bwowulila nga olina byoyisemu nga byamugaso eri embooziyaffe nga tolina buzibu kubitubulira, oliwaddembe okubitubulira. Mubufunze, singa tugenda kumulamwa, nolaba nti gwe oba omuntu omulala gwomanyi yayita mumbeera eyo, tujja kukukweyanza singa otubulirako bwebyali naye sikyateka nti olina okubitubulira.
3. Tekyetagisa kumala kuwanika mukono okwogera. Yogerera mukifo wotudde naye wa ekitibwa entesa yabalala. Okukubaganya ebirowoozo kuno kugenda kutwala sawa biri. Waliwo omuntu yena wano atasobola kubeerawo kumala banga eryo lyetugenda okumala nga tukubaganya ebirowoozo? Waliyo alina ekibuuzo nga tetunaba kutandika?

(Twagala kuba nga tukwata amaloboozi gemboozi yaffe. Ebikwatidwa kumalobozi bituyamba kulaba nti tuwulira buli kimu ekyogedwa tusobole okubiwandiika byona. Abo boka abakola kukunonyereza kuno bebajja okuwulira kumaloboozi gano oba okusoma kubiwandikidwa. Nga okunonyereza kuwedde nokutekateka ebiwandikidwa awamu, obutambi buno bujja kwononebwa. Waliwo atandiyagadde kukwatibwa maloboozi?)

**Thank you for your attention, we are now set to begin the discussion**

***(mwebale nnyo okumpuliliza katugende mmaaso n’okubaganya ebilowozo?)***

**Section A: Beginning Questions**

1. How easy is it for people to access HIV testing services in this community?

(*Omuntu ayanguyirwa kyenkanaki okufuna empeereza yo’kwekebeza akawuka kasilimu mukitundu kino?)*

1. If people test HIV-positive, what challenges do such people face in accessing HIV treatment? How can these challenges be minimized? (*Singa omuntu asangibwa nga alina akawuka kasirimu, asanga kusomozebwaki mukusobola okufuna obujanjabi bwakawuka kasirimu? Okusomozebwa okwo kusobola kumalibwawo kutya?)*

**Section B: Perceptions of HIV Self-testing**

HIV self-testing is a way of seeing if you have contracted HIV by conducting the test yourself, instead of having to see a doctor and ask for a test. Special kits let you swab your gums to collect some saliva, and then test the saliva to see if there’s any sign of HIV in your body. You can do this entirely by yourself in the privacy of your own home, and it takes about 20-30 minutes to complete.

*Enkola oyokwekebela akawuka kasirimu yengeri omuntu gyayinza okumanya nti okwatiddwa akawuka kasirimu nga yekebela yekanayekka mukiffo kyokulaba musawo okumukebela. Ebikozesebwa ebyenjawulo bikusobozesa okukozesa akati ayisibwa kubibuno okusobola okufuna amarusu era oluvanyuma nokebera okulaba oba waliwo akabonero kona akalaga akawuka kasirimu mumubirigwo. Kino osobolela ddala kukyekolela nga olimukiffo ekyekyama gamba nga mumakaago era nga kitwaala edakiika 20-30 zokka.*

1. What are your general impressions about HIV self-testing? How willing will people in your community be to use HIV self-test kits to test for HIV? Please explain your response.

*Ensonga yabantu okwekebeza akawuka kasilimu bokanabokka ogyogelakoki? Abantu bomukitundukyo banettanila kyenkanaki okukozesa enkola eno? Nkusaba onnyonyole ensongazo.*

1. What kind of support would people need before they conducted the self-test? What kind of support would they need after conducting the self-test?

*Abantu betaaga kubudabudibwa/kuyambibwa batya ngatebanaba kwekebela kawuka kasilimu?*

*Abantu betaaga kubudabudibwa/kuyambibwa batya ngabamalilizza kwekebela kawuka kasilimu?*

1. In your opinion, would it be better if people self-tested alone or if they self-tested with someone around them? Please explain your response.

*Mundowoozayo, kisingako singa omuntu yekebela nga aliyekka oba nga waliwo nomuntu omulala? Nkusaba onnyonyole ensongazo.*

1. In your opinion, should HIV self-test kits be given to all people in the community or to selected people? If you had free HIV self-test kits to distribute in the community: a) which people or groups of people would you prioritize? b) which people would you deny kits? Please explain your response.

Mundowoozayo, ebikozesebwa mukwekebela akawuka kasilimu biweebwe buli muntu yenna *mukitundukyo oba biweebwe abantu abalondemu? singa obadde olina okugaba ebikozesebwa mukwekebela akawuka kasimu kubwelele mu kitundukyo; a) bantuki oba bibiinaki ebya’bantu byewandilonzemu okuwa? b) bantuki botandiwadde bikozesebwa bino? Nkusaba onnyonyole ensongazo.*

1. Should HIV self-test kits be denied to young people who are not yet of age (i.e. below 18 years)? If young people were given HIV self-test kits, how should they be supported to use them to test for HIV?

*Abaana abataneetuuka gamba’baliwansi wemyaka 18 baganibwe okuweebwa ebikozesebwa mukwekebela akawuka kasilimu? Singa abaana abataneetuuka bawebwa ebikozesebwa mukwekebela akawuka kasilimu, bayinza kuyambibwa batya okubikozesa okwekebela akawuka kasilimu?*

1. If HIV self-tests became available in this community, where would people be comfortable to obtain them?

*Singa ebikozesebwa okwekebela akawuka kasilimu biletebwa mukitundu kino, abantu bandyaagadde bifunilawa?* ***(Probe for: health facility, VHT, friend, peer educator, Beach Management Unit, etc.)***

1. What fears or concerns do you have in general regarding HIV self-testing?

*Okutwaliza wamu, olina bwelalikivuki oba biki byotidde kubikwata kumuntu okwekebela akawuka kasilimu yekkanayekka****.***

**Section C: *Perceptions of community-based HIV self-testing distribution***

1. If HIV self-test kits became freely in the community, how comfortable would you be to obtain them from a member of your community?

*Singa ebikozesebwa mukwekebela akawuka kasilimu bifuuse byabwelele, kyandikwanguyidde kyenkanaki okubifuna kumuntu owomukitundukyo?*

1. Which kinds of people would be comfortable obtaining kits from? What qualities should they have?

*Bantu kikaaki abandikwanguyidde kufunako ebikozesebwa mukwekebela akawuka kasilimu?*

*Bandibadde nabisanyizoki?*

1. What challenges do you envisage with community-based distribution of HIV self-test kits? How can these challenges be minimized?

*Buzibuki kwolengela mumaaso munkola eyokugaba ebikozesebwa mukwekebela akawuka kasilimu mubyaalo? Obuzibu buno buyinza kukendezebwa/kumalibwaawo butya?*

1. How can confidentiality be ensured in a setting where people obtain kits from a member of their community rather than at the health facility?

*Ebyaama binakuumibya bitya munkola eyokuba nti abantu bafuna ebikozesebwa bino okuva eli munnaabwe kukitundu kyaabwe sossi okuva kuddwaliro?*

***Section D: Social Network Structures***

1. What social groupings of: a) young people and b) adult men exist in this community?

*Bibiinaki ebyokutabagana; a) ebya’bavubuka, b) abasajja abakulu ebiri mukitundukino?*

1. How many members do such groupings have? How do members join these groupings? Who is eligible to join the grouping? How often do members exit the groupings?

*Ebibiina byoyogedeko bilina abantu bamekka? Abantu fafuuka batya bamemba mubibiina bino? Ani asaanidde okwetaba mubibiina bino?* ***(Probe for membership aspects for groups of young men and groups of adult men separately.)***

1. How often do such groupings meet? Where do they meet? How are their meetings organized?

*Ebibiiba bino bisisinkana buli ddi? Bisisinkana wa? Enkiiko zaabwe baziteekateeka batya?*

1. What benefits do: a) young people and b) adult men obtain from belonging to such groupings?

*Birungiki a) abavubuka b) abasajja abakulu byebafuna mukubeela ekitundi oba gamba mamemba mubibiina bino?*

1. How many people in this meeting belong to social network groupings in this community? Bantu *Bameka kumwe abalimubibiina bino byokutabagana ebili mukituundu kino?*

***(Nsaba mwongele okumbulila kubibiina bino byemulimu.)***

1. If we wanted to distribute HIV self-test kits to members of your groupings, how best would this be done? Who would lead the distribution exercise? What qualities would such a person have?

*Singa tubadde twagala okugaba ebikozesebwa mukwekebela akawuka kasilimu eli bamemba bebibiina byamwe, tuyinza kukikola tutya? Ani ayinza okkulembelamu okugaba ebikozesebwa bino? Omuntu ono yandibadde nabisaanyizoki?*

**Section E: Perceptions and Suggestions on Network-based, Peer-led HIV Self-testing**

(Ettendekeero ly’ebyobulamu erya Makareere University School of Public Health nga likolaganila wamu nekitongole kya Rakai Health Sciences Program (nga eno yetiimu yabanonyereza) Era nga aba Fogarty International Centre naba Africa Research Excellence Fund bebatadde sente mukunonyereza kuno nekilubirirwa ekyokutekawo enkola eyokugaba ebikozesebwa mukwekebela akawuka kasirimu kubwerere eri abantu bomukitundu kino okusobola okwekebela akawuka kasirimu boka naboka nga tekibetagisiza kusooka kugenda muddwalilo. Ebikozesebwa bino bijakubatusibwako bantu bannamwe mukitundu kino. Abantu bano bajja kulondebwa tiimu y’abanonyereza benyini nga bamaze kwebuuza kubakulembeza bamwe mukitundukino, era baja kutendekebwa mukwekebera akawuka kasirimu era nemukusoma ebyo ebiba bivudde mukwkebela. Buli anaba atendekedwa ajakussemba abantu abalala 20 eli tiimu y’abanonyereza (Abo abasembeddwa bateekedwa okamanyibwa abo ababasebye, nga muno mwotwalidde emikwano, bebakola nabo, abafamile, oba abo abali mubibiina ebyokumanyagana). Okusobobala okukirizibwa okuwebwa ebikozesebwa. Olokubanti tetulina bikozesebwa kwebeka bimala kuntandikwa yeteekateeka eno, abantu 10 bokka ku 20 abanaasembebwa era ngabalina ebisaanyizo ebibakkiriza okwetaba mukunonyereza bebajja okufuna ebikozesebwa. Buli omu kwabo ekumi abanaaba balondedwa okuwebwa ebikozesebwa bino bajja kutendekebwa munkozesa yabyo era nemukutaputa ebyo ebiba bivudde mukwekebela bokanaboka. Abo abanesanga nga balina akawuka kasirimu bajjakuzibwamu amanyi okugenda maaso okulaba nga bakozesa olimu oba amalwaliro gombi agakolela awamu naffe mukunonyereza kuno basobole **okukkakasa ebyo ebivudde mukwekebela bokanaboka**. Singa kizuulibwa nti balina akawuka kasilrimu bajjakuyungibwa kubujajjabi bwa kawuka kasirimu amangu ddala ng’eteeka lyokukebela ela nokujanjaba bweligamba elyekitongole kya gavumenti eky’ebyobulamu. Ekigendererwa kyenteekateeka eno kwekutumbula okukebela kwakawuka kasirimu era nokuyungibwa kubujanjabi bwakawuka kasirimu eri abavubuka abali wakati wemyaka (15 ku 24) era nabasajja abakuze okuva (kumyaka 25+) nokudda wagulu ngababeela Kasensero kumwalo nga tubanjulila enkola eyokwekebela akawuka kasirimu nga tekibetagisa kusoka kugenda muddwaliro.)

1. What are your initial thoughts about such a program? If you had the opportunity, would you have liked to be part of this program? Why or why not?

*(Enteekateeka eno ogyilowoozako ki? Singa obadde olina omukisa wandyagadde okubeela mu nteekateeka eno? Lwaki oba lwaki nedda?)*

1. How comfortable would people in this community be in obtaining HIV self-test kits from a peer- leader who has been trained to distribute HIV self-test kits?

*(Abantu bomukitundu kino bayinza kukitwala batya singa bafuna ebikozesebwa mukwekebela akawuka kasirimu okuva eli omukulembeze wabwe atendekedwa okibisasaanya mukitundu kyabwe?)*

1. What benefits do you envisage in implementing such a program in this community? How can these benefits be maximized?

*(Miganyulwoki gyosubila singa enteekateeka eyengeli eno etekebwa mukitundukyo? Emiganyulwo egyo giyinza kutukibwako gyitya?)*

1. What challenges do you envisage in the implementation of this program in this community? How can these challenges be minimized?

*(Okosomozebwaki kwosubila singa enteekateeka eyengeli eno etekebwa mukitundukyo? Okusomozebwa kuno kuyinza kumalibwawo kutya?)*

1. If you were asked to select someone who should be trained to distribute HIV self-test kits to young people aged 15 – 17 years in this community, what qualities would you look for?

*(Singa obadde osabiddwa okulonda omuntu alina okutendekebwa okusasaanya ebikozesebwa mukwekebela akawuka kasirimu eli abavubuka abali wakati we’myaka 15 – 17 mukitundu kino, bisanyizoki byewanditunulidde?)*

1. If you were asked to select someone who should be trained to distribute HIV self-test kits to young people aged 18 – 24 years in this community, what qualities would you look for?

*(Singa obadde osabiddwa okulonda omuntu alina okutendekebwa okusasaanya ebikozesebwa mukwekebela akawuka kasirimu eli abavubuka abali wakati we’myaka 18 – 24 mukitundu kino, bisanyizoki byewanditunulidde?)*

1. If you were asked to select someone who should be trained to distribute HIV self-test kits to adult men (25 years or older) in this community, what qualities would you look for?

*(Singa obadde osabiddwa okulonda omuntu alina okutendekebwa okusasaanya ebikozesebwa mukwekebela akawuka kasirimu eli abasajja abakulu okuva kumyaka 25 no’kuddawagulu mukitundu kino, bisanyizoki byewanditunulidde?*

1. How would confidentiality be ensured in a program that distributes HIV self-test kits through trained local distributors?

*(Okukuuma ebyaama kunaakakasibwa kutya munteekateeka eyokusasaanya ebikozesebwa mukwekebela akawuka kasilimu okuyita mwabo abatendekedwa okkola amulimu egwo mubitundu byamwe.)*

1. If you were one of those individuals who have been selected to receive HIV self-test kits from a trained local HIV self-test kits distributor, would you prefer that you receive the kit from a same-sex distributor or it does not matter? Where (venues) would you prefer to receive the kits from? Why those particular places?

*(Singa obadde omu kwabo abalondendwa okufuna ebikozesebwa mukwekebela akawuka kasirimu okuva ewoyo atendekedwa okubisasanya, wandyagadde okufuna empereza eno okuva kumuntu bwemufananya obutonde oba ekyo sikikulu? Wandyagadde kufunilawa ebikozesebwa bino? Lwaki oyagala kubifunila mukiffo ekyo?)*

1. Individuals who self-test for HIV are usually advised to seek confirmatory HIV testing at a government health facility. In your opinion, would individuals who self-test for HIV accept to seek confirmatory HIV testing? How can more individuals be encouraged to seek confirmatory HIV testing?

*Abantu abeekebela akawuka kasilimu bokanabokka bulijjo baweebwa amagezi okunda mudwalliro lya gavumenti okulaba nga beekebeza okusobola okukkakasa ebyo byebafunye mukwekebela bokanabokka. Mundowoozayo, abantu abeekebedde akawuka kasiliimu bokanabokka bayinza okukkiriza okugende okukkakasa ebyo byebafunye mukwekebela? Tuyiinza tutya okulaba nga twogela kumuwendo gwa’bantu abagenda maaso nokukkakasa ebyo byebafunye mukwekebela akawuka kasilimu bokanabokka?*

1. In general, how would this kind of program be improved to reach more people in the fishing communities?

*(Okutwaliza awamu enteekateeka eno eyinza kutumbulwa etya okusobola okutuusibwa eri abantu abawelako ababeela kumyalo?)*

**THANK YOU FOR YOUR TIME**
