# Supplementary material for: Feasibility and acceptability of a pilot, peer-led HIV self-testing intervention in a hyperendemic fishing community in rural Uganda
Source: PLoS One. 2020 Aug 7;15(8):e0236141. doi: 10.1371/journal.pone.0236141 (PMC7413506; doi:10.1371/journal.pone.0236141)
Supplement: Attachment — Submitted filename: Review for Plos 1.docx [file pone.0236141.s006.docx]

Dear Authors,

I found this paper interesting .Below I summarize my main points, in the hope that you might nonetheless find them constructive.

**Comments:**

1. The introduction is missing the significance of the study, the gaps in the literature that the study is filling, who stand to benefit from this work, how is it pushing the streams of the literature, how can this results be replicated into policy actions based on its novelty

2. We know that any new innovation is likely to be accepted mainly driven by curiosity – the authors may want to explain more broadly on what might be driving factors of uptake of HIVST in this region

3. The study is susceptible to both social desirability bias and non-response bias considering that the data collection strategy queried sensitive and potentially stigmatizing information. For example, HIV status, linkage to ART ect. The authors may want to explain some possible measures that were taken to prevent self-reported biases.

In conclusion, I recommend a revision to factor in these 3 points comprehensively.

Best wishes,
Reviewer
